# Maternal Serum Catestatin Levels in Gestational Diabetes Mellitus: A Potential Biomarker for Risk Assessment and Diagnosis

**DOI:** 10.3390/jcm14020435

**Published:** 2025-01-11

**Authors:** Nazan Vanli Tonyali, Gulsan Karabay, Burak Arslan, Gizem Aktemur, Betul Tokgoz Cakir, Zeynep Seyhanli, Busra Demir Çendek, Seval Yilmaz Ergani, Hasan Eroglu, Sumeyye Mermi, Şevki Celen

**Affiliations:** 1 Department of Perinatology, Ankara Etlik City Hospital, Ankara 06170, Türkiye; drgulsankarabay@gmail.com (G.K.); drgizemkizilbuga@gmail.com (G.A.); btltkgz@gmail.com (B.T.C.); drzeynepseyhanli@gmail.com (Z.S.); dr.svl7@gmail.com (S.Y.E.); sevki.celen@sbu.edu.tr (Ş.C.); 2 Department of Psychiatry and Neurochemistry, Institute of Neuroscience & Physiology, The Sahlgrenska Academy at the University of Gothenburg, 42130 Mölndal, Sweden; burak.arslan@gu.se; 3 Department of Obstetrics and Gynecology, Ankara Etlik City Hospital, Ankara 06170, Türkiye; dr.busra_demir@hotmail.com (B.D.Ç.); sumyyeaslan@gmail.com (S.M.); 4 Department of Perinatology, Faculty of Medicine, Afyon Kocatepe University, Afyon 03204, Türkiye; drhasaner@gmail.com

**Keywords:** gestational diabetes mellitus, catestatin, biomarkers, glucose tolerance, pregnancy outcomes

## Abstract

**Background/Objectives:** Gestational diabetes mellitus (GDM) presents significant risks for both maternal and neonatal health, affecting fetal growth and increasing the likelihood of future diabetes mellitus (DM) development in affected women. The dysregulation of metabolic biomarkers, including catestatin, has been implicated in GDM pathophysiology. However, the clinical significance of catestatin in GDM remains poorly understood, particularly in the context of different therapeutic approaches. **Methods:** This observational, prospective, and cross-sectional study was conducted to evaluate maternal serum catestatin levels in gestational diabetes mellitus (GDM) patients and healthy controls. Data were collected at a single time point during the second trimester of pregnancy (24 to 28 weeks). Participants were categorized based on their glucose tolerance and GDM management strategies (diet regulation or insulin therapy). **Results:** Receiver Operating Characteristic (ROC) analysis demonstrated the diagnostic significance of serum catestatin levels in GDM, suggesting a cut-off value of >9.61 ng/mL for discriminating between women with and without GDM. However, further research is needed to elucidate the mechanistic role of catestatin in GDM and its utility in guiding therapeutic interventions. **Conclusions:** Our study highlights the potential of catestatin as a biomarker for GDM risk stratification and monitoring, complementing existing diagnostic tools. Integrating metabolic biomarkers like catestatin into clinical management approaches may optimize maternal and neonatal health outcomes in GDM. However, the limitations of our study, including its cross-sectional design and sample size, underscore the need for future multicenter studies to validate our findings comprehensively.

## 1. Introduction

Gestational diabetes mellitus (GDM) can cause irregular fetal growth, early delivery, preeclampsia, and cesarean section. Its prevalence ranges from 3% to 7% depending on race and geography [1,2]. A systematic review in Turkey found GDM prevalence ranging from 1.9% to 27.9% with a combined rate of 7.7%. The highest prevalence was in the Black Sea Region (17.6%) and the lowest was in central Anatolia (5.1%) [3]. Elevated blood glucose levels during pregnancy are associated with these adverse outcomes and increase the likelihood of developing diabetes mellitus (DM) later in life [4].

Chromogranin A (CgA) is a peptide that is cleaved by a proteolytic enzyme to produce catestatin (CST). Adrenergic neurons and adrenal chromaffin cells both store and release CgA in addition to catecholamines [5]. Catestatin, a 21-amino-acid protein, suppresses catecholamine release by interacting with neuronal nicotinic acetylcholine receptors and reduces hypertension [6]. This peptide is found in endocrine organ secretory granules, including pancreatic insulin-producing P and glucagon-producing A cells [5,7]. CST improves insulin sensitivity, lowers inflammation, and inhibits catecholamine release [7].

GDM has been linked to various biomarkers, including adiponectin, leptin, and C-reactive protein (CRP) [8]. These biomarkers play roles in inflammation, insulin sensitivity, and glucose metabolism. However, CST has unique advantages due to its dual anti-inflammatory and insulin-sensitizing properties, directly targeting the underlying mechanisms of GDM pathophysiology. Unlike other biomarkers, CST not only inhibits catecholamine release but also improves insulin sensitivity and reduces systemic inflammation [9]. Because of these effects, CST has a major role in regulating glucose metabolism, especially in diseases like type 2 diabetes and metabolic syndrome, which are characterized by high levels of insulin resistance. Researchers are looking at the therapeutic potential of CST supplementation as a way to address these underlying mechanisms of inflammation and insulin resistance in order to improve metabolic health [10]. Low CST levels may predispose to hypertension and metabolic diseases. Some studies have shown that CST has an insulin-sensitizing effect [11]. Studies in mice with CgA deficiency demonstrated that CST treatment inhibited hepatic glucose production. Scholars have shown that administering CST reduces the amount of adipose tissue, promotes the breakdown of fats in adipose tissue, and enhances the uptake and breakdown of fatty acids in the liver of these animals [12]. While decreased CST levels have been linked to type 2 diabetes in adults, there is no research on CST and GDM in pregnant women. Currently, CST kits are available from specialty suppliers at a cost of approximately 3–8.3 USD (United States Dollars),making CST a potentially cost-effective alternative to traditional screening methods. In our clinic, GDM is diagnosed with a two-stage approach with 50 g GTT followed by 100 g GTT applied to positive cases, and the cost of each test is approximately USD 3–4.5. Beyond direct costs, these tests require patients to make multiple hospital visits and incur indirect costs such as lost time, work interruptions, and patient discomfort from non-serious side effects. Additionally, due to misinformation campaigns targeting glucose challenge tests in Turkish media, GTT screening rates decreased from 7.55% in 2013 to 3.9% in 2015 [13]. This decline may lead to an increase in undiagnosed cases of GDM and related complications. Given these challenges, the investigation of alternative biomarkers such as CST is of great importance to improve diagnostic accuracy and cost-effectiveness, especially in high-risk populations.

Our hypothesis was that maternal serum CST levels would differ significantly between GDM and normal pregnancies, supporting its potential as a novel GDM biomarker. This study examines CST levels in the second trimester, comparing GDM patients’ 100 g OGTT results with those of normal OGTT patients. We aimed to assess CST’s clinical utility in GDM risk assessment and diagnosis while acknowledging the need for future studies to explore its potential role in treatment guidance.

## 2. Materials and Methods

The Etlik City Hospital Perinatology Clinic conducted this prospective study from January to November 2020. After briefing, participants consented in writing. The study was approved by the hospital ethics committee (AEŞH-EK1-2023-475) and adhered to the Helsinki Declaration.

Patients were diagnosed with GDM using the American College of Obstetricians and Gynecologists (ACOG) and the International Association of Diabetic Pregnancy Working Groups (IADPSG) standards [14]. Between 24 and 28 weeks of pregnancy, the 50 g oral glucose tolerance test (OGTT) examined all subjects for GDM. In individuals with a 1 h glucose level of 140 mg/dL or higher in 50 g OGTT, 100 g was used after 8 h of fasting. The diagnostic criteria were based on the American Diabetes Association recommendations: fasting blood glucose levels over 95 mg/dL, 1 h blood glucose levels below 185 mg/dL on the 100 g OGTT, 2 h blood glucose levels above 155 mg/dL, and 3 h blood glucose levels below 140 mg/dL. Two or more high glucose levels at these criteria were needed to diagnose GDM. The right attention and therapy were given to pregnant women with GDM.

These were the inclusion criteria: Participants were 18–40-year-old women pregnant with one child who gave informed permission. The study excluded women with diabetes, gastrointestinal problems, inflammatory bowel disease, autoimmune disorders, recent probiotic or antibiotic usage, fetal abnormalities, or intrauterine growth restriction.

Every subject underwent a thorough history and clinical assessment after informed consent. Maternal serum CST levels (measured in the second trimester at GDM diagnosis), dietary information and adherence, insulin therapy details (type, dose, and frequency), and GDM diagnosis details (age, BMI, parity, gestational age, and obstetric history) were collected. Three milliliters of serum from control and GDM blood samples obtained between 24 and 28 weeks were put into Eppendorf tubes.

Blood glucose levels divided GDM patients into two follow-up groups: insulin treated and diet only. To assess CST levels, the diet-only regulated blood glucose group (A1GDM), the insulin-regulated blood glucose group (A2GDM), and the control group were statistically compared. The composite of adverse neonatal outcomes consisted of any of the following: Apgar score < 5 at 5 min, ventilator support within 24 h of birth, preterm birth (<37 weeks), or low birth weight (<2500 g).

Analysis of CT Levels: Maternal serum CST levels were measured using a commercially available enzyme-linked immunosorbent assay (ELISA) kit (Human Catestatin ELISA Kit, MyBioSource, San Diego, CA, USA). Three milliliters of serum samples were collected in the morning after an overnight fast from both the control and GDM groups between 24 and 28 weeks of gestation. The serum was separated by centrifugation and stored at −80 °C until analysis.

Statistical analysis: Statistical analysis was performed using SPSS version 25. Descriptive statistics were utilized for demographic and clinical variables. Continuous variables were expressed as mean ± standard deviation or median with interquartile range (IQR) based on their distribution, while categorical variables were presented as percentages. For normally distributed continuous variables, ANOVA with Tukey post hoc correction was applied, whereas non-normally distributed continuous variables were analyzed using the Kruskal–Wallis or Mann–Whitney U tests. Categorical variables were compared using Chi-square or Fisher’s exact tests where appropriate.

An independent samples *t*-test was used to compare the CST levels between groups with Levene’s test employed to verify the homogeneity of variances and adjust results when necessary. Logistic regression analysis was performed to identify factors associated with CNO and GDM, while receiver operating characteristic (ROC) analysis was conducted to assess the discriminatory capacity of CST levels. The ROC analysis included calculations for area under the curve (AUC), cut-off points, sensitivity, and specificity. A *p*-value of <0.05 was considered statistically significant throughout the analysis.

Sample size calculation: G*Power software 3.1.9.7 was used to calculate the sample size. Based on a ratio of 1:1:1 between the three groups, an alpha error of 0.05, 80% power, and an estimated minimum sample size of 14 patients per group was needed. More samples were obtained for our study than we had anticipated. A total of 87 patients were enrolled in the trial. The study compared CST levels across three groups: 32 patients with normal glucose tolerance, 36 patients with diet-regulated GDM, and 19 patients with insulin-regulated GDM.

## 3. Results

Table 1 provides a summary of the pregnant women’s clinical findings, laboratory data, and demographic information. No statistically significant differences were found in BMI (*p* = 0.840), parity (*p* = 0.076), or gestational age (*p* = 0.747) between the groups. However, the maternal age in the A2GDM group was higher.

An independent samples *t*-test was conducted to evaluate the relationship between maternal serum CST levels and the presence of a history of GDM in previous pregnancies (Table 2). Group statistics showed that the mean CST level was 14.22 ± 10.99 ng/mL in women without a history of GDM (n = 78) and 8.65 ± 1.47 ng/mL in women with a history of GDM (n = 9). Levene’s test indicated unequal variances (F = 7.920, *p* = 0.006), so the results were interpreted using the assumption of unequal variances. The *t*-test revealed a statistically significant difference in CST levels between women with and without a history of GDM (t(83.407) = 4.160, *p* < 0.001). The mean difference in CST levels was 5.56 ng/mL (95% CI: 2.90 to 8.22 ng/mL). These results suggest that women with a history of GDM in previous pregnancies have significantly lower CST levels compared to those without such a history.

Similarly, an independent samples *t*-test was conducted to evaluate the relationship between maternal serum CST levels and body mass index (BMI) measured at the time of serum collection. Participants were divided into two groups based on their BMI: less than 30 (n = 41) and 30 or greater (n = 46). Group statistics revealed that the mean CST level was 16.48 ± 13.24 ng/mL for the BMI < 30 group and 11.11 ± 6.54 ng/mL for the BMI ≥ 30 group. Levene’s test indicated unequal variances (F = 18.708, *p* < 0.001), so results were interpreted using the assumption of unequal variances. The *t*-test showed a statistically significant difference in CST levels between the two BMI groups (t(56.878) = 2.357, *p* = 0.022). The mean difference in CST levels was 5.38 ng/mL (95% CI: 0.81 to 9.95 ng/mL). These results suggest that women with a BMI < 30 have significantly higher CST levels compared to those with a BMI ≥ 30.

There was no difference between the control group and the A1GDM group or between the A1GDM and A2GDM groups despite a considerable disparity in the groups’ histories of GDM in prior pregnancies. There was no difference between A1GDM and A2GDM, but there was a difference between the control group and the two GDM groups for family history of diabetes. When it came to polyhydramnios, the control group and the A2GDM group differed significantly from each other but neither the A1GDM nor the A2GDM groups differed from the control group. There was a statistically significant difference (*p* < 0.001) between the two GDM groups and the control group when CST levels were taken into account. CST levels were greater in the GDM patient group compared to the control group (Table 3).

Logistic regression analysis showed no significant association between composite neonatal outcomes (CNO) and birth weight, polyhydramnios, or CST levels in either univariate (*p* > 0.1) or multivariate analysis (*p* > 0.2). These findings indicate that the evaluated variables did not independently predict CNO in this cohort (Table 4).

The ROC analysis used in this investigation to assess the relevance of serum CST level in GDM is displayed in Table 3. With a *p* value of 0.002, the serum CST level’s AUC was 0.705 (95% CI: 0.587–0.822). A cut-off value of >9.61 ng/mL discriminates women with 65.6% sensitivity and 76.4% specificity.

There was no significant difference in serum CST level between the A1GDM and A2GDM groups (AUC = 0.513, 95% CI: 0.348–0.679, *p* = 0.873).

Both the control and GDM groups showed a statistically significant negative connection between maternal serum CST levels and 50 g OGTT results (Spearman’s rho = −0.305, *p* = 0.004, n = 87).

In our analysis, hierarchical logistic regression was applied to evaluate the independent effect of CST on GDM while controlling for known risk factors, including maternal age, nulliparity, BMI, family history of diabetes, and the presence of polyhydramnios. The initial model, which included these basic risk factors, showed a Nagelkerke R^2^ of 0.240. When CST was added in the second model, the Nagelkerke R^2^ increased to 0.320, indicating improved explanatory power. CST was found to have a statistically significant and independent association with GDM risk (OR = 0.934, 95% CI: 0.883–0.988, *p* = 0.017). This suggests that for each unit increase in CST levels, the odds of GDM decrease by about 1.07 times. Other variables, such as maternal age, nulliparity, BMI, and polyhydramnios, were not significantly associated with GDM in the final model (*p* > 0.05). These results highlight the potential role of CST as an independent biomarker for GDM risk assessment (Table 5).

## 4. Discussion

The findings of this study shed light on the intricate interplay between metabolic biomarkers, clinical parameters, and pregnancy outcomes in GDM. By comparing CST levels among healthy pregnant women and those with GDM managed through different therapeutic modalities, this study aimed to contribute new insights into the pathophysiology and management of this prevalent pregnancy complication.

Oxidative stress (OS) plays a pivotal role in the pathophysiology of GDM through mechanisms involving the adrenergic system. Studies indicate that OS increases catecholamine release by activating adrenergic receptors, which in turn exacerbate inflammation and cardiovascular dysfunction [15]. The development of OS and the subsequent process contribute to insulin resistance and glucose intolerance, which are the main features of GDM. Additionally, OS impairs endothelial function, inhibiting nitric oxide production and leading to vascular dysfunction [16]. CST, one of the peptides formed by the breakdown of CgA, provides vasodilation by inhibiting catecholamine release [17]. Proteins such as Chromogranin B (CgB) also have effects that attenuate OS-induced damage by regulating mitochondrial function and reducing reactive oxygen species [18]. These findings suggest that the interplay between adrenergic signaling, OS, and endothelial dysfunction plays a fundamental role in understanding the pathophysiology of GDM and may offer opportunities for new biomarkers and therapeutic targets.

GDM is associated with pregnancy and an increased risk of preeclampsia, macrosomia, and preterm birth [19]. And beyond pregnancy outcomes, it also significantly increases the long-term risk of type 2 diabetes and cardiovascular disease for the mother as well as the risk of obesity and metabolic syndrome for the child [20]. These findings underscore the multifaceted clinical impacts of GDM and highlight the need for comprehensive risk assessment and management strategies to mitigate both short-term and long-term adverse outcomes.

Our analysis revealed that healthy controls had greater CST levels than GDM patients regardless of therapy approach. This finding underscores the potential dysregulation of CST secretion associated with insulin resistance and impaired glucose metabolism, which are both characteristic of GDM. While previous studies have reported alterations in CST levels in various metabolic disorders, including type 2 diabetes mellitus (T2DM), our study extends this knowledge to the specific context of GDM. Dasgupta et al.’s study highlights CST’s potential in alleviating obesity-induced insulin resistance [9]. Their findings indicate that CST mitigates inflammation and proinflammatory macrophage infiltration, thereby reducing hepatic insulin resistance and ER stress. These results support our observation of low CST levels in GDM patients being associated with insulin resistance and metabolic dysfunction. The anti-inflammatory effects of CST could be beneficial in GDM management.

According to our research, maternal serum CST levels were considerably lower in women with a history of GDM than in those without (14.22 ± 10.99 ng/mL vs. 8.65 ± 1.47 ng/mL, *p* < 0.001). Similarly, women with a BMI < 30 had significantly higher CST levels than those with a BMI ≥ 30 (16.48 ± 13.24 ng/mL vs. 11.11 ± 6.54 ng/mL, *p* = 0.022). Simunovic et al. (2019) found lower serum CST concentrations in obese children and adolescents than healthy controls [21]. In the study by Pankova et al., it was reported that CST levels were significantly lower in hypertensive and diabetic patients and showed a negative correlation with insulin resistance (HOMA-IR) (r = −0.481, *p* < 0.001) [11]. Similarly, the study by Lu et al. demonstrated a strong association between BMI and diabetes risk, which was partially mediated by lipid metabolism [22]. Our study further underscores the potential role of metabolic biomarkers, such as catestatin, in understanding the pathophysiology of diabetes. These findings suggest that obesity-associated inflammation and insulin resistance may contribute to the suppression of CST levels, potentially disrupting its protective effects. However, the observation that the protective effects of CST may be diminished in individuals with obesity and insulin resistance suggests that these findings require validation in larger and more diversified populations.

Furthermore, our analysis revealed differences in pregnancy outcomes among the study groups. While birth weight did not differ significantly between groups, the incidence of preterm birth and polyhydramnios was higher in the A2GDM group. Importantly, the CNO did not differ substantially between the groups, indicating that maternal hyperglycemia management may have a greater impact on neonatal health outcomes than CST levels alone. The analysis revealed no significant association between CNO and variables such as birth weight, polyhydramnios, or CST levels in both univariate and multivariate logistic regression. Neonatal outcomes are affected by complex interactions of multiple factors such as maternal hyperglycemia, placental dysfunction, and preterm birth. In this study, it was observed that birth weight, polyhydramnios and CST levels did not have an independent effect on CNO. It can be thought that CST regulates mostly maternal metabolic parameters but has a limited effect on neonatal outcomes. This supports the idea that neonatal complications can be minimized by controlling GDM.

ROC analysis identified a serum CST cut-off value of >9.61 ng/mL for distinguishing GDM with 65.6% sensitivity and 76.4% specificity. While CST effectively differentiates healthy pregnancies from those with GDM, it does not distinguish between A1GDM and A2GDM (AUC = 0.595, 95% CI: 0.457–0.723, *p* = 0.208, *p* = 0.208, *p* = 0.208). These findings suggest CST is useful for GDM risk assessment but not for severity differentiation. In this study, logistic regression analysis identified family history of diabetes and maternal serum CST levels as significant factors associated with GDM. A family history of diabetes notably increased the likelihood of GDM (Exp(B) = 5.68, 95% CI: 1.60–20.13, *p* = 0.006). Additionally, each unit increase in maternal serum CST level was associated with a 6% decrease in GDM risk (Exp(B) = 0.94, 95% CI: 0.88–0.99, *p* = 0.017). These findings underscore the multifactorial nature of GDM, where genetic predisposition and metabolic biomarkers like CST play critical roles. While the identified cut-off value demonstrates diagnostic potential, its validation in independent and diverse populations is essential to confirm its generalizability and clinical utility. Future studies should focus on evaluating this cut-off across varied demographic and clinical settings to reduce the risk of false-positive and false-negative results.

CST inhibits catecholamine release, reduces inflammation, and regulates metabolic processes, making it a possible clinical biomarker for hypertension, heart failure, and diabetes [23]. This study has shown that levels of CST are changed in individuals with cardiovascular illnesses and metabolic syndrome. Our findings suggest that CST levels could help differentiate normal pregnancies from GDM and assist in risk stratification and diagnosis.

The negative correlation between catestatin levels and OGTT findings across the research population, including control and GDM groups, shows that catestatin may modulate glucose metabolism during pregnancy. The inverse relationship between catestatin levels and glucose tolerance suggests CST’s insulin-sensitizing effects, which are consistent with studies demonstrating its role in glucose metabolism under metabolic stress conditions [24]. Additionally, animal studies have demonstrated that CST can inhibit hepatic glucose production, reduce inflammation, and enhance insulin sensitivity, underscoring the importance of adequate CST levels for effective glucose regulation [24,25]. Given these findings, CST supplementation may be a therapeutic option in diabetes diseases, particularly GDM, where metabolic abnormalities may compromise CST action. Although CST is a promising biomarker and therapeutic agent for GDM, there are some practical challenges. The high cost of diagnostic kits, limited access to advanced technologies, and differences in CST levels due to individual factors may affect diagnostic accuracy and clinical applicability. These limitations require the development of cost-effective and accessible methods and the investigation of the heterogeneity of CST in different populations. Future studies should focus on improving the clinical application of CST.

Our study has some limitations, including the inability to infer causality due to its cross-sectional design and the limited sample size from a single center, which may restrict the generalizability of the findings. Larger multicenter studies are needed to validate our results and identify additional variables.

## 5. Conclusions

Our study highlights the potential role of metabolic biomarkers, such as CST, in the risk assessment and diagnosis of GDM. While CST levels were associated with glycemic control and general pregnancy outcomes, no direct impact on specific complications, such as preterm birth or polyhydramnios, was demonstrated. These findings suggest that CST levels could contribute to a more targeted approach in GDM management, aiding in identifying high-risk cases and tailoring risk assessment strategies. Future studies, including randomized controlled trials, are needed to further explore the utility of CST in gestational diabetes.

## Figures and Tables

**Table 1:** Pregnancy characteristics of the study population.

Variable(s)	A1GDM (n = 36)	A2GDM (n = 21)	Control (n = 33)	*p*	Post Hoc Comparisons *p* Value		
					A GDM 1-A2 GDM	A1 GDM-Control	A2 GDM-Control
Age years	30.8 ± 5.7	34.3 ± 4.6	30.5 ± 5.7	**0.046 ^a^**	0.095	NA	**0.049**
BMI kg/m^2^	30.3 ± 5.3	32.4 ± 4.6	29.5 ± 3.1	0.840 ^a^			
Gravida n	3.06 (1–10)	3.58 (1–6)	2.78 (1–7)	0.076 ^b^			
GA weeks	178.6 ± 7.7	179.4 ± 6.2	177.8 ± 7.3	0.747 ^a^			
C/S	9 (25)	5 (23.8)	11 (34.4)	0.671 ^c^			
History of GDM	5 (13.9)	4 (21.1)	0 (0)	**0.038 ^c^**	NA	**0.029**	**0.007**
Family DM History	17 (47.2)	13 (68.4)	5 (15.6)	**0.001 ^c^**	NA	**0.002**	**0.001**

A1GDM: diet-controlled gestational diabetes, A2GDM: insulin controlled gestational diabetes, DM: diabetes mellitus, GA: gestational age, BMI: body mass index, C/S: cesarean section, n: number, kg: kilogram, m^2^: square meter. A *p* value of <0.05 indicates a significant difference. Statistically significant *p*-values are in bold. ^a^: ANOVA test (post hoc test: Tukey correction), ^b^: Kruskal–Wallis test (post hoc test Bonferroni), ^c^: Chi-square (post hoc test: pairwise Chi-square test).

**Table 2:** Independent samples *t*-test results for catestatin levels based on history of GDM and BMI.

	n	Mean (ng/mL)	SD (ng/mL)	*p*
History of GDM				
No	78	14.22	10.99	<0.001
Yes	9	8.65	1.47	
BMI				
<30	41	16.48	13.24	0.022
≥30	46	11.11	6.54	

GDM; gestational diabetes, BMI; body mass index, SD; standard deviation, n; number.

**Table 3:** The laboratory values and pregnancy outcomes of the study groups.

	A1GDM (n = 36)	A2GDM (n = 21)	Control (n = 33)	*p*	Post Hoc Comparisons *p* Value		
					A1GDM-Control	A2GDM-Control	A1GDM-A2GDM
50 g GGT mg/dL (one-hour blood sugar)	176.5 ± 24.1	179.8 ± 26.8	108.7 ± 40.2	**<0.001 ^a^**	**<0.001**	**<0.001**	NA
Hba1c %	5.5 (4.7–6.1)	5.9 (5.3–7.5)	-	**0.004 ^b^**			
Birth weight g	3287.6 ± 62.9	3117.2 ± 96.2	3077.3 ± 104.7	0.22 ^a^			
Apgar 1	9 (8–9)	9 (7–9)	9 (7–9)	0.89 ^c^			
Apgar 5	10 (9–10)	10 (8–10)	10 (8–10)	0.89 ^c^			
Catestatin ng/mL	8.7 (2.8–49.9)	8.6 (5.7–42.3)	13.5 (6.2–48.1)	**0.021 ^c^**	**0.014**	**0.032**	NA
Preterm birth	2 (6.2)	3 (8.3)	1 (5.3)	0.89 ^d^			
Polyhydramnios	2 (5.6)	5 (26.3)	1 (3.1)	0.006 ^d^	NA	0.041	0.022
CNO	2 (6.1)	6 (16.6)	1 (4.7)	0.265 ^d^			

Hba1c hemoglobin a1c; GTT, glucose tolerance test; mg, milligram; dL, deciliter; ng, nanogram; CNO, composite adverse neonatal outcome. Data are expressed as mean ± SD, median and minimum–maximum or number (percentage) where appropriate. A *p* value of < 0.05 indicates a significant difference. Statistically significant *p*-values are in bold. ^a^: ANOVA test (post hoc test: Tukey), ^b^: Mann–Whitney U test, ^c^: Kruskal–Wallis (post hoc test Bonferroni), ^d^: Chi-square (post hoc test: pairwise Chi-square test).

**Table 4:** Logistic regression analysis for factors associated with composite neonatal outcome.

	Univariate LR		Multivariate LR
	OR (95% CI)	*p*	*p*
Birth weight g *	0.973 (0.936–1.012)	0.167	0.208
Polyhydramnios	1.738 (0.182–16.566)	0.631	0.999
Catestatin *	0.892 (0.722–1.102)	0.291	0.536

* These parameters were treated as a continuous variable.

**Table 5:** Results of logistic regression analysis of factors associated with gestational diabetes.

Variable	Univariate LR		Multivariate LR Model 1		Multivariate LR Model 2	
	OR (95% CI)	*p*	OR (95% CI)	*p*	OR (95% CI)	*p*
Maternal Age *	1.051 (0.972–1.137)	0.213	1.039 (0.945–1.144)	0.428	0.985 (0.885–1.096)	0.780
Nulliparity	0.903 (0.350–2.327)	0.832	1.548 (0.490–4.885)	0.456	1.558 (0.465–5.220)	0.472
BMI *	1.083 (0.977–1.201)	0.129	1.065 (0.949–1.194)	0.284	1.056 (0.939–1.189)	0.363
Family History of Diabetes	6.482 (2.17–19.31)	**0.001**	5.684 (1.848–17.482)	**0.002**	6.690 (1.986–22.532)	**0.002**
Polyhydramnios	4.521 (0.530–38.556)	0.168	2.903 (0.273–30.889)	0.377	6.605 (0.360–121.048)	0.203
Catestatin *	0.944 (0.902–0.989)	**0.014**	-	-	0.934 (0.883–0.988)	**0.017**

* These parameters were treated as a continuous variable. In model 1, catestatin was not included in the model. Statistically significant *p*-values are in bold.

## Data Availability

The data presented in this study are available on request from the corresponding author.
